# Association of metabolic syndrome with carotid atherosclerosis in low-income Chinese individuals: A population-based study

**DOI:** 10.3389/fcvm.2022.943281

**Published:** 2022-08-19

**Authors:** Changqing Zhan, Qiao Wang, Zongsheng Chen, Hongbo Pang, Jun Tu, Xianjia Ning, Jinghua Wang, Shizao Fei, Xuelei Ji

**Affiliations:** ^1^Department of Neurology, Wuhu No. 2 People's Hospital, Wuhu, China; ^2^Department of Pathology, Wuhu No. 2 People's Hospital, Wuhu, China; ^3^Department of Neurology, Tianjin Medical University General Hospital, Tianjin, China; ^4^Laboratory of Epidemiology, Tianjin Neurological Institute, Tianjin, China; ^5^Tianjin Neurological Institute, Key Laboratory of Post-Neuroinjury Neuro-Repair and Regeneration in Central Nervous System, Ministry of Education and Tianjin City, Tianjin, China; ^6^Department of Endocrinology, Wuhu No. 2 People's Hospital, Wuhu, China

**Keywords:** atherosclerosis, carotid plaque, total plaque area, metabolic syndrome, components

## Abstract

**Background:**

This study aimed to explore the association of the presence and number of components of metabolic syndrome (MetS) with carotid atherosclerosis by measuring the presence of carotid plaque and total plaque area (TPA) in a population from a low-income area with high incidence of stroke of northern China.

**Methods:**

A cross-sectional study was conducted in a rural area of Tianjin, China from April 2014 to January 2015. The presence of plaque and TPA measurement was determined by carotid ultrasound. The presence and number of components of MetS was ascertained using the modified International Diabetes Federation criteria for the Asian population.

**Results:**

Among a total of 3,583 individuals aged ≥ 45 years, the overall prevalence of MetS was 54.5%. MetS and its components were related to the presence of carotid plaque as well as TPA. Multivariate analysis showed MetS was associated with a 20% higher risk of carotid plaque presence (95% confidence interval: 1.01, 1.42; *P* = 0.036) and an 18% increase in TPA (95% confidence interval: 0.08, 0.27; *P* < 0.001). The number of MetS components showed an increasing trend with the risk of carotid plaque presence and increased TPA. Among single components of MetS, high BP accounted for the largest proportion and was an independent risk factor of carotid plaque and increased TPA.

**Conclusions:**

Among individuals aged 45 years or more, we confirmed MetS and its components were associated with carotid atherosclerosis in a low-income population of northern China. The presence of MetS and a higher number of MetS components exacerbated the risk of carotid atherosclerosis; among the five MetS components, high blood pressure was associated with the greatest risk. Targeted atherosclerosis prevention and intervention should include identification and treatment of MetS, especially high blood pressure.

## Introduction

A study of global stroke burden showed that there were 80.1 million prevalent cases of stroke with 5.5 million deaths in 2016 (1). Although the global age-standardized mortality rate decreased by 36.2% from 1990 to 2016, the economic burden remains significant (1). In America, the total annual direct medical costs related to stroke will increase from $7.155 billion in 2012 (2). In China, the annual absolute medical cost of stroke in 2013 surpassed that of the United States, reaching $ 7.77 billion (3). As the most populated country with a rapidly aging population, reducing the morbidity and mortality of stroke had become an increasingly serious challenge in China.

The most common subtype of stroke in China is ischemic stroke, accounting for 69.6% of all strokes (3), and carotid atherosclerotic plaque rupture is an important cause of ischemic stroke (4). Ultrasound measurement of carotid plaque is considered to be a surrogate endpoint for predicting stroke events in epidemiological studies (5, 6). Measurement of carotid plaque burden by determining the total plaque area (TPA) is regarded as a stronger predictor of cerebrovascular risk compared with the use of common carotid artery intima-media thickness (7, 8). Therefore, early detection and management of occurrence and progression of carotid plaque may reduce morbidity and mortality of stroke, especially for individuals with high-risk factors.

Metabolic syndrome (MetS) is a cluster of three or more risk factors for cardiovascular disease, including hypertension, obesity, and dyslipidemia. The aggregation of these risk factor components of MetS are related with progression of atherosclerosis (9) and increased stroke risk (10, 11). Various studies have shown that individuals with MetS have a higher risk of carotid plaque prevalence and higher TPA compared with non-affected individuals (12, 13). Moreover, the risk of carotid atherosclerosis increased with a higher number of MetS components (12, 13). However, most previous studies were conducted in more developed countries or in high-income urban areas in China (14–16), whereas few studies have been conducted in a low-income population, especially those aged 45 years or more.

Therefore, the aim of this study was to explore the association of the presence and number of MetS components with carotid atherosclerosis (carotid plaque and TPA) among a low-income population aged 45 years or older in northern China.

## Methods

### Participants and study design

This population-based cross-sectional study was conducted in rural areas of Tianjin, China from April 2014 to January 2015. The population and research data came from the Tianjin Brain Study, which has been described previously (17). In brief, 14,251 participants from 18 administrative villages in rural Tianjin, China were included; low-income farmers accounted for approximately 95%, with a per capita disposable income of <1600 US dollars in 2014 (18). Our study previously demonstrated that a high incidence of stroke in rural areas of Tianjin with age-standardized incidence of first-ever stroke per 100,000 person-years up to 318.2 in 2012 and the incidence of stroke increased annually by 6.5% overall from 1992–2012 (19). The current study recruited all local permanent residents aged ≥ 45 years using the methods of clustering sampling. Those individuals without histories of cardiovascular and cerebrovascular diseases were included, and those who underwent carotid endarterectomy or carotid stenting were excluded. All eligible participants completed a questionnaire, including questions on lifestyle factors and medical treatment; underwent physical examination and carotid artery ultrasound screening; and provided blood samples for biochemical measurements.

This study was approved by the Ethics Committee at Tianjin Medical University General Hospital and conformed to the Declaration of Helsinki regarding the use of human subjects. Written informed consent was obtained from each patient for this study.

### Data collection

Data collection was performed *via* face-to-face interviews by trained epidemiological researchers using a pre-specified questionnaire. Demographic information, including name, sex, age, and educational level, were obtained from previous records. Given the high risk of stroke in young and middle-aged residents in this population based on previous studies (19, 20), and based on the age composition of the study population, all participants were separated into three age groups: 45–54, 55–64, and ≥65 years...., focusing on this age group is particularly meaningful. According to the length of formal education of China, educational level was divided into three groups: illiteracy (without education), elementary school (1–6 years), and above elementary school (>6 years). The medical history included previous diagnosis of diabetes, hypertension, hyperlipidemia, and corresponding medication history. Lifestyle factors included history of cigarette smoking (defined as smoking more than one cigarette per day for at least 1 year, divided into never smoking, and current or ever smoking) and alcohol consumption (defined as drinking more than 500 g of alcohol per week for at least 1 year, divided into never drinking, and current or ever drinking).

### Physical examination and biochemical tests

Physical examination included waist circumference (cm), body mass index (BMI, kg/m^2^), and blood pressure (BP; mmHg). The specific measurement process has been previously described (21). All blood samples were collected with the participants in the fasting state. Fasting plasma glucose (FPG) and serum total cholesterol (TC), triglyceride (TG), high-density lipoprotein cholesterol (HDL-C), and low-density lipoprotein cholesterol (LDL-C) concentrations were measured at the Ji County People's Hospital. Moreover, Carotid ultrasonography and 12-lead echocardiography were also conducted by a professional. Body mass index (BMI) was calculated as weight (kg) divided by the square of height (m^2^).

### Ultrasonography measurements

Bilateral carotid images were obtained by trained and certified technicians who were blinded to participants' details using B mode ultrasonography (Terason 3000; Burlington, MA, US) with a 5–12 MHz linear array transducer. A carotid artery plaque was defined as a focal area protruding into the vessel lumen that was at least 50% thicker than the adjacent intima-media thickness (22). Radiologists evaluated the presence and extent of plaque in extracranial carotid artery trees (common carotid artery, the bifurcation, internal and external carotid arteries) bilaterally. Plaque area was measured by tracing the perimeter of a plaque in a longitudinal section: the perimeter of the largest plane of the plaque and TPA (mm^2^) was determined as the sum of plaque area across all segments between the clavicle and the angle of the jaw. Images were obtained and digitally stored according to a standard protocol (22).

### Criteria for metabolic syndrome

MetS was defined according to the modified International Diabetes Federation criteria for the Asian population in which individuals had to fulfill ≥3 of the following criteria (23): (1) abdominal obesity (waist circumference ≥ 90 cm in men and ≥80 cm in women), (2) elevated TG (TG ≥ 1.70 mmol/L or on drug treatment for hypertriglyceridemia), (3) low HDL-C (HDL-C < 1.03 mmol/L in men and <1.3 mmol/L in women, or specific treatment for this lipid abnormality), (4) high BP (≥130/85 mmHg) or on antihypertensive medication, (5) elevated FPG (≥5.6 mmol/L) or on antidiabetic treatment.

The MetS components refer to the aforementioned five diagnostic parameters (abdominal obesity, elevated TG, low LDL-C, high BP, and elevated FPG), and the number of components that individuals of MetS had was divided into three groups: 1–2, 3–4, and 5 components.

### Statistical analysis

The Kolmogorov-Smirnov test was performed to identify the pattern of data distribution. Continuous variables are presented as the mean ± standard deviation for normally distributed variables (age, BMI, WC, systolic BP, diastolic BP, FPG, TC, TG, HDL-C, LDL-C) and median (interquartile range [IQR]) for non-normally distributed variables (TPA). TPA exhibited positive skewness and was natural log (ln)-transformed to achieve normality. Categorical variables (sex, age group, education level, smoking status, alcohol consumption, MetS presence and its components and number of components) are presented as frequencies and percentages. Group comparisons were performed using the independent sample *t*-test (for normally distributed data) or Mann-Whitney test (for non-normally distributed data). The chi-squared test was used for correlations between categorical variables and for inter-group analysis. Logistic regression analysis was used to determine the effects of MetS and its components on the presence of plaque and presented as odd ratios (OR) and 95% confidence intervals (CI). A multivariate linear regression model was used to estimate the effects of MetS and its components on ln-transformed TPA and presented as the β-coefficient and 95% CI. For ln-transformed TPA, the regression coefficient provides the percentage change in the dependent variable for each 1-unit change in the independent variable (13). A two-tailed *P*-value of < 0.05 was considered as statistically significant. All statistical analyses were performed using SPSS software (version 25.0; SPSS, Chicago, IL, USA).

## Results

**Figure 1:**
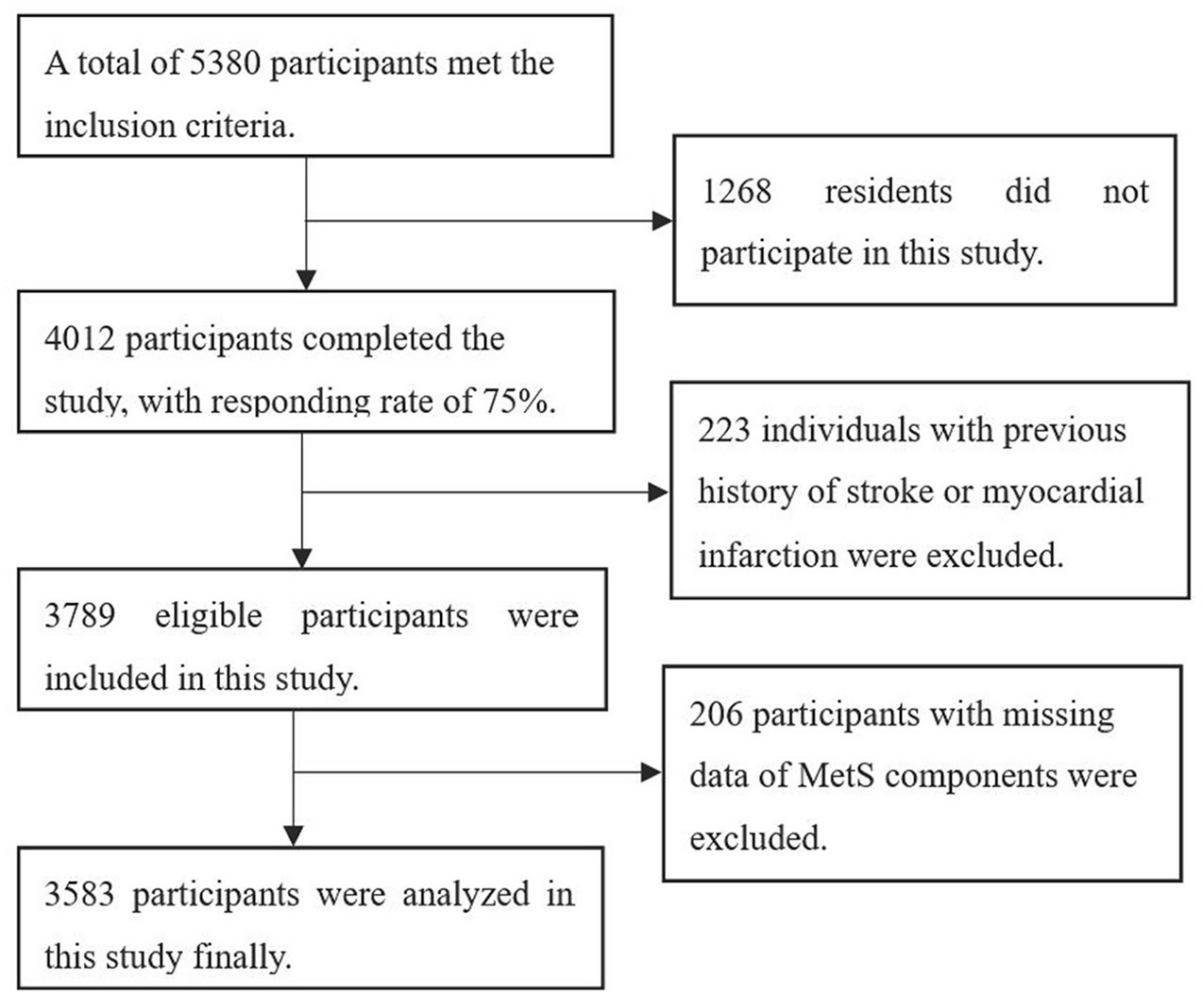
Flowchart of participants.

A total of 4,012 residents participated in this survey among 5,380 residents ≥ 45 years old. The responding rate was 75%. After excluding 223 residents with histories of cardiovascular and cerebrovascular diseases and 206 individuals with missing data of MetS components. 3,583 participants were included in this study finally (Figure 1).

### Demographic characteristics

Table 1 shows the characteristics of the 3,583 participants, including 1,482 (41.4%) men and 2101 (58.6%) women. The mean age was 60.06 ± 9.52 years. More than 60% of individuals were illiterate or had only elementary education. The number of individuals with never smoking were three times greater than the number with current or ever smoking. In men, 57% were current or ever smokers, and 36.1% were current or ever alcohol drinkers. In addition, 29% of individuals used anti-Hypertensive drugs, whereas few used a lipid-lowering agent. The average level of BMI, WC, FBG, TC, TG, HDL-C, LDL-C, systolic BP, and diastolic BP were 25.53 kg/m^2^, 89.15 cm, 5.92 mmol/L, 4.86 mmol/L, 1.75 mmol/L, 1.46 mmol/L, 2.70 mmol/L, 146.53 mmHg, and 86.84 mmHg, respectively.

**Table 1:** Characteristics of study participants by sex.

**Category**	**Men**	**Women**	**Total**
Total	1482 (41.4)	2101 (58.6)	3583 (100)
Age, means (SD), years	61.32 (9.70)	59.17 (9.30)	60.06 (9.52)
Age group, *n* (%)			
45~54 years	386 (26.0)	734 (34.9)	1120 (31.3)
55~64 years	587 (39.6)	823 (39.2)	1410 (39.4)
≥65 years	509 (34.3)	544 (25.9)	1053 (29.4)
Education level, *n* (%)			
0 years	134 (9.0)	498 (23.7)	632 (17.6)
Elementary school	672 (45.3)	932 (44.4)	1604 (44.8)
Above elementary school	676 (45.6)	671 (31.9)	1347 (37.6)
Smoking status, *n* (%)			
Never smoking	638 (43.0)	2050 (97.6)	2688 (75.0)
Now or ever smoking	844 (57.0)	51 (2.4)	895 (25.0)
Alcohol consumption, *n* (%)			
Never drinking	947 (63.9)	2072 (98.6)	3019 (84.3)
Now or ever drinking	535 (36.1)	29 (1.4)	564 (15.7)
Medication history, *n* (%)
Anti-hypertensive	431 (29.1)	607 (28.9)	1038 (29.0)
Anti-diabetes	72 (4.9)	124 (5.9)	196 (5.5)
Lipid lowering agent	8 (0.5)	11 (0.5)	19 (0.5)
Presence of CP, *n* (%)	744 (50.2)	752 (35.8)	1496 (41.8)
TPA, median (IQR), mm^2^	18.40 (23.10)	12.45 (15.76)	15.04 (19.38)
BMI, means (SD), Kg/m^2^	25.15 (3.44)	25.80 (3.8)	25.53 (3.69)
WC, means (SD), cm	89.35 (8.92)	89.01 (9.19)	89.15 (9.08)
FBG, means (SD), mmol/L	5.91 (1.41)	5.93 (1.67)	5.92 (1.57)
TC, means (SD), mmol/L	4.62 (1.01)	5.03 (1.09)	4.86 (1.08)
TG, means (SD), mmol/L	1.59 (1.19)	1.87 (1.24)	1.75 (1.23)
HDL-C, means (SD), mmol/L	1.40 (0.43)	1.51 (0.47)	1.46 (0.46)
LDL-C, means (SD), mmol/L	2.62 (1.22)	2.76 (1.29)	2.70 (1.26)
SBP, means (SD), mmHg	147.95 (21.36)	145.53 (22.59)	146.53 (22.12)
DBP, means (SD), mmHg	88.54 (11.23)	85.64 (11.41)	86.84 (11.43)

SD, standard deviation; SBP, systolic blood pressure; DBP, diastolic blood pressure; BMI, body mass index; WC, Waist circumference; FBG, fasting blood glucose; TC, total cholesterol; TG, triglycerides; HDL-C, high density lipoprotein cholesterol; LDL-C, low density lipoprotein cholesterol; CP, carotid plaque; TPA, total plaque area.

Mean and standard deviation (SD) is used for normally distributed values.

Median and interquartile range (IQR) is used for non-normally distributed values.

The prevalence of carotid plaque was 41.8% overall, 50.8% in men, and 35.8% in women. The median TPA was 15.04 mm^2^ (IQR: 19.38) overall, 18.40 mm^2^ (IQR: 23.10) in men, and 12.45 mm^2^ (IQR: 15.76) in women.

### Characteristics of MetS and its components

Table 2 shows that the total prevalence of MetS was 54.5% overall, 63.2% in women, and 42.3% in men. Participants with 0–2 and 3–4 MetS components accounted for 45.5 and 46.1% respectively, while individuals with 5 MetS components accounted for just 8.5%. In women, more than half of individuals had 3–4 components, while in men, individuals with 0–2 components comprised the highest percent. Among all MetS components, the most common components were high BP (81.5%) and abdominal obesity (70.9%) overall, while low HDL-C (28.7%) was the least common component in both men and women.

**Table 2:** Characteristics of MetS and its components.

**Category**	**Men**	**Women**	**Total**
MetS, *n* (%)			
Yes	627 (42.3)	1327 (63.2)	1954 (54.5)
No	855 (57.7)	774 (36.8)	1629 (45.5)
Num. of MetS components, *n* (%)			
0–2	855 (57.7)	774 (36.8)	1629 (45.5)
3–4	551 (37.2)	1099 (52.3)	1650 (46.1)
5	76 (5.1)	228 (10.9)	304 (8.5)
MetS components, *n* (%)			
Raised BP	1254 (84.6)	1665 (79.2)	2919 (81.5)
Raised FPG	783 (53.6)	1021 (49.4)	1804 (51.1)
Reduced HDL-C	264 (18.1)	748 (36.2)	1012 (28.7)
Raised TG	448 (30.6)	875 (42.4)	1323 (37.5)
Abdominal obesity	723 (48.9)	1811 (86.4)	2534 (70.9)

### Relationship between MetS and carotid plaque presence

Table 3 shows some risk factors associated with the presence of carotid plaque in the univariate analysis; these factors included sex, age, education level, smoking status, MetS components (abdominal obesity, high BP, elevated FPG, low HDL-C, all *P* < 0.05), BMI, TC, and LDL-C levels. There was not a correlation between carotid plaque and MetS presence or the number of MetS components in the univariate analysis.

**Table 3:** Associated factors of carotid plaque presence and TPA in the univariate analysis.

**Risk factors**	**CP**, ***n*** **(%)/means (SD)**		***P*-value**	**TPA** **median (IQR)/β (95%CI)**	***P*-value**
	**No**	**Yes**			
Sex			<0.001		<0.001
Women	1349 (64.2)	752 (38.5)		12.45 (15.76)	
Men	738 (49.8)	744 (50.2)		18.40 (23.10)	
Age group			<0.001		<0.001
45~54 years	861 (76.9)	259 (23.1)		10.21 (10.52)	
55~64 years	784 (55.6)	626 (44.4)		14.71 (16.94)	
≥65 years	442 (42.0)	611 (58.0)		18.91 (25.91)	
Education			<0.001		0.039
0 years	327 (51.7)	305 (48.3)		16.00 (23.83)	
Elementary school	881 (54.9)	723 (45.1)		15.64 (20.88)	
Above elementary school	879 (65.3)	468 (34.7)		14.22 (15.82)	
Smoking status			<0.001		<0.001
Never smoking	1632 (60.7)	1056 (39.3)		14.08 (19.02)	
Now or ever smoking	455 (50.8)	440 (49.2)		16.95 (21.96)	
Alcohol consumption			0.81		0.154
Never drinking	1774 (58.8)	1245 (41.2)		14.88 (19.26)	
Now or ever drinking	313 (55.5)	251 (44.5)		16.06 (20.61)	
MetS			0.102		0.179
Yes	1119 (57.3)	835 (42.7)		15.29 (20.29)	
No	968 (59.4)	661 (40.6)		15.12 (19.00)	
Single components of MetS					
Raised BP	1621 (55.5)	1298 (44.5)	<0.001	15.64 (20.49)	<0.001
Raised FPG	999 (55.4)	805 (44.6)	<0.001	15.81 (21.04)	0.007
Reduced HDL-C	619 (61.2)	393 (38.8)	0.014	14.59 (17.22)	0.807
Raised TG	764 (57.7)	559 (42.3)	0.333	14.90 (19.35)	0.986
Abdominal obesity	546 (52.4)	495 (47.6)	<0.001	14.37 (18.70)	0.033
Num. of components of MetS			0.424		0.395
<3	968 (59.4)	661 (40.6)		15.12 (19.00)	
3–4	946 (57.3)	704 (42.7)		15.35 (20.61)	
5	173 (56.9)	131 (43.1)		14.31 (17.14)	
LDL-C	2.42 (1.02)	3.08 (1.45)	<0.001	0.02 (−0.01, 0.05)	0.240

The results of the multivariate logistic regression analysis in Table 4 show the presence of MetS (OR: 1.20, 95% CI: 1.01, 1.42), 3–4 MetS components (OR = 1.21, 95% CI: 1.02, 1.44) and 5 MetS components (OR = 1.47, 95% CI: 1.09, 1.98) were independent risk factors of carotid plaque presence (all *P* < 0.05), after adjustment for age, sex, education, and LDL-C level. Among the five MetS components, only high BP (OR = 1.49, 95% CI: 1.21, 1.83) was associated with carotid plaque presence. Furthermore, there was an obviously increasing trend wherein an increasing number of MetS components significantly elevated the risk of carotid plaque: compared with <3 components, the risk increased by 21 and 47% for 3–4 and 5 MetS components, respectively.

**Table 4:** Relationship between metabolic syndrome and carotid atherosclerosisin multivariate analysis.

**Risk factors**	**ln (TPA)**		**Carotid plaque**		
	**β (95%CI)**	**P**	**References**	**OR (95%CI)**	**P**
MetS	0.18 (0.08, 0.27)	<0.001	No	1.20 (1.01, 1.42)	0.036
Num. of components of MetS			<3		
3–4	0.16 (0.07, 0.26)	0.001		1.21 (1.02, 1.44)	0.030
5	0.28 (0.12, 0.44)	0.001		1.47 (1.09, 1.98)	0.011
MetS components					
Raised BP	0.19 (0.07, 0.31)	0.002	No	1.49 (1.21, 1.83)	<0.001
Raised FPG	0.06 (−0.03, 0.14)	0.173	No	1.05 (0.90, 1.22)	0.559
Reduced HDL–C	0.05 (−0.04, 0.15)	0.276	No	1.06 (0.89, 1.26)	0.530
Raised TG	0.06 (−0.03, 0.15)	0.184	No	1.14 (0.96, 1.34)	0.128
Abdominal obesity	0.06 (−0.01, 0.23)	0.054	No	0.86 (0.69, 1.06)	0.152

MetS, Metabolic syndrome; FBG, fasting blood glucose; BP, blood pressure; TG, triglycerides; HDL–C, high density lipoprotein cholesterol.

Multivariate analysis in MetS, Num. of MetS components and MetS components were adjusted by age, education, smoking status, LDL–C.

### Relationship between MetS and TPA

Table 3 presents the correlations between sex, age, education level, smoking status, MetS components (abdominal obesity, high BP, elevated FPG), BMI, and TPA in our total sample (all *P* < 0.05). We did not find correlations between TPA and MetS or with the number of MetS components in univariate analysis.

After adjustment for age, sex, education, smoking, alcohol consumption, LDL-C, and BMI, the presence of MetS (β = 0.18; 95% CI: 0.08, 0.27; *P* < 0.001), high BP (β = 0.16; 95% CI: 0.07, 0.26; *P* = 0.001), 3–4 MetS components (β = 0. 0.16; 95% CI: 0.07, 0.26; *P* = 0.001), and 5 MetS components (β = 0.28; 95% CI: 0.12, 0.44; *P* = 0.001) were associated with risk of a greater area of carotid plaques in multivariate linear regression analysis. We also found that MetS was associated with increased TPA, which was in accordance with the carotid plaque results. Moreover, individuals with 5 MetS components exhibited the greatest risk of a larger TPA: compared with <3 components, ln-transformed TPA increased by 16 and 28% for 3–4 and 5 MetS components, respectively.

## Discussion

In this population-based study of 3,583 individuals aged ≥ 45 years from low-income rural areas of Tianjin, China, we found that individuals with MetS had a tendency toward a higher risk of carotid plaque presence and increased TPA compared to those without MetS. Furthermore, an increasing number of MetS components was associated with an aggravated risk of carotid atherosclerosis. Among all components of MetS, high BP accounted for the largest proportion in our population and was an independent risk factor of carotid plaque and TPA. As expected, the risk of carotid atherosclerosis increased with MetS and the number of its components.

Previously, a cross-sectional study of an urban population in Hangzhou, China, showed MetS and the number of its components were associated with an increased prevalence of carotid plaque (14). Moreover, a cohort study with a 5-year follow-up in a community dwelling population (*n* = 1257) demonstrated that MetS was significantly associated with the presence of carotid plaque and positively increased risks of myocardial infarction, stroke, and cardiovascular disease mortality independently of traditional cardiovascular disease risk factors in older Chinese adults (24). In the ongoing prospective Ansan Geriatric Study of a population-based cohort, it was reported that MetS was associated with the progression of early carotid atherosclerosis in participants aged at least 60 years and as a risk predictor of newly developed carotid plaque, with a hazard ratio of 1.916 (95% CI: 1.059, 3.466; *P* = 0.031) (25). Similar to the results of prior studies, we found that MetS was associated with carotid plaque presence, and the risk of carotid plaque presence increased by 20, 21, and 47% for <3, 3–4, and 5 MetS components, respectively. In contrast, one study found that MetS was not independently related to the presence of carotid plaque (15).

In the present study, we also measured TPA as an indicator of carotid plaque burden, which provided additional information concerning the degree of carotid atherosclerosis. Research shows that TPA is a better indicator for predicting the risk of atherosclerotic disease when compared with using carotid intimal media thickness (26, 27). Moreover, a cross-sectional study of 972 non-diabetic patients showed that the association between MetS and the burden of atherosclerosis was independent from other traditional vascular risk factors and that TPA increased by 37% in individuals with MetS (β = 0.37, 95% CI: 0.12, 0.63, *P* = 0.004) (28). This is similar to the present results, with an 18% higher risk of increased TPA with MetS presence (β = 0.18, 95% CI: 0.08, 0.27, *P* < 0.001). Furthermore, a trend was observed in the present findings toward higher TPA as the number of components of MetS increased. This has been less frequently reported in previous studies; however, one review concluded that measurement of carotid plaque burden was useful for risk stratification, research into the genetics and biology of atherosclerosis, and measuring effects of therapy on atherosclerosis (29). We hold great expectations toward TPA measurement to identify plaque progression in individuals at high risk of stroke and believe that closer attention should be given to the role of TPA measurement.

Among the five components of MetS, high blood pressure is a major risk factor for the development of atherosclerosis (30). In the present study, high BP was the most frequent MetS component and was the largest contributor to carotid atherosclerosis. The results are consistent with a study reported in Japan (16). We found that high BP was an independent risk factor of carotid plaque and increased TPA, while the other four components were not significantly related to carotid atherosclerosis. These results are in agreement with reports from the Trømso study (31) and Cyprus Study (13), both of which reported that high BP was the only MetS component consistently associated with both carotid plaque presence and increased TPA. As expected, our findings also support high BP as a traditional risk factor for cardiovascular disease and stroke and suggest that high BP plays an important role in the atherosclerotic progression.

Consistent with the findings of other studies, not all components involved in MetS showed an independent association with carotid atherosclerosis in the multivariate analysis (32, 33). The heterogeneity of different study populations and sample sizes may affect the association between specific component of MetS and carotid atherosclerosis. However, it is clear that the risk of adverse cardiovascular events in individuals with a cluster of multiple cardiometabolic risk factors is far greater than in those with a single risk factor (34, 35). This is a reminder of the importance of early identification of and intervention with individuals with multiple risk factors to reduce the risk of adverse cardiovascular events. Moreover, both MetS and CIMT are age-related diseases (36, 37). Previous studies have shown that with increasing age, the CIMT gradually thickens (38) and the risk of MetS increases (39). Similar to previous research findings, in this study, the prevalence of CP and TPA increased with increasing age group.

Our study has some limitations. First, this was a cross-sectional study, thus we cannot make any causal inferences about the role of the MetS and its components on carotid atherosclerosis. Further longitudinal observations will be needed to address this relationship. Second, we focused on only individuals living in a township in Tianjin, China, which may limit the applicability of our results to other populations. Third, our study population included only participants aged ≥ 45 years and therefore these findings are not generalizable to broader age groups. Nonetheless, given the high risk of stroke in our population based on previous studies (19, 20), focusing on this age group is particularly meaningful.

## Conclusion

This study focuses on the low-income population in Tianjin, China, and conducts a real-world study. This study confirmed that the presence and number of components of MetS was associated with increased risk of carotid plaque presence and increased TPA in a low-income population over 45 years of age. Among the five MetS components, high BP accounted for the highest proportion and was an independent risk factor of carotid atherosclerosis. As there is a rapidly aging population and increased incidence of stroke in low-income areas (19), detailed screening and management of individuals with MetS, especially toward effective BP control, may significantly reduce the risk for atherosclerotic progression.

## Data availability statement

The raw data supporting the conclusions of this article will be made available by the authors, without undue reservation.

## Ethics statement

The studies involving human participants were reviewed and approved by the Ethics Committee at Tianjin Medical University General Hospital. The patients/participants provided their written informed consent to participate in this study.

## Author contributions

XJ, SF, and JW were involved in conception and design, critical review in for this article, and data interpretation for this article. XN was involved in data analysis for this article. CZ and QW were involved in manuscript drafting. CZ, QW, ZC, HP, and JT were involved in data collection, case diagnosis, and confirmation for this article. All authors contributed to the article and approved the submitted version.

## Funding

This study was supported partly by Science and Technology Panning Project of Wuhu city (No. 2021YF66).

## Conflict of interest

The authors declare that the research was conducted in the absence of any commercial or financial relationships that could be construed as a potential conflict of interest.

## Publisher's note

All claims expressed in this article are solely those of the authors and do not necessarily represent those of their affiliated organizations, or those of the publisher, the editors and the reviewers. Any product that may be evaluated in this article, or claim that may be made by its manufacturer, is not guaranteed or endorsed by the publisher.
